# Gastrointestinal Bleeding Is Associated with Worse In-Hospital Outcomes in Patients with ST-Segment Elevation Myocardial Infarction: An Analysis of the National Inpatient Sample

**DOI:** 10.3390/jcm15187140

**Published:** 2026-09-14

**Authors:** Shreyas Ranganath, Trishna Parikh, Adishwar Rao, Rohan Patil, Ria Shah, Ishan Gupta, Eshwar Ranganath, Jeff Kue, Alberto Bueso-Perez, Aarohi Parikh, John Pina, Thomas Etheridge, Richard Johnson, Lori Varma, Venkat Keshav Chivukula, Bela Patel, Bindu Akkanti

**Affiliations:** 1 McGovern Medical School at the University of Texas Health Science Center at Houston, 6431 Fannin St., Houston, TX 77030, USA; rangans@ccf.org (S.R.); rohan.patil@tuhs.temple.edu (R.P.); eshwar.ranganath@uth.tmc.edu (E.R.); jeff.t.kue@uth.tmc.edu (J.K.); alberto.j.buesoperez@uth.tmc.edu (A.B.-P.); 2 Department of Internal Medicine, Case Western Reserve University/University Hospitals of Cleveland, Lakeside Building, 3rd Floor Suite, 11100 Euclid Avenue, Cleveland, OH 44106, USA; john.pina@uhhospitals.org; 3 Department of Internal Medicine, Guthrie Robert Packer Hospital, 1 Guthrie Sq., Sayre, PA 18840, USA; adishwar.rao@guthrie.org; 4 Rice University, 6100 Main Street, Houston, TX 77005, USA; ria.shah@rice.edu; 5 Arizona College of Osteopathic Medicine, Midwestern University, 19555 59th Ave, Glendale, AZ 85308, USA; igupta@honorhealth.com; 6 Department of Internal Medicine, HCA Healthcare—Kingwood/University of Houston College of Medicine, 22999 US 59 N, Kingwood, TX 77339, USA; parikhaarohi.md@gmail.com; 7 Department of Internal Medicine, McGovern Medical School at the University of Texas Health Science Center at Houston, 6431 Fannin St., MSB 1.150, Houston, TX 77030, USA; thomas.h.etheridge@uth.tmc.edu (T.E.); richard.g.johnson@uth.tmc.edu (R.J.); 8 University of Texas at Dallas, 800 W Campbell Rd., Richardson, TX 75080, USA; lori.varma@gmail.com; 9 Advanced Cardiopulmonary Therapies and Transplantation, McGovern Medical School at the University of Texas Health Science Center at Houston, 6431 Fannin St., MSB 1.150, Houston, TX 77030, USA; venkat.keshav.chivukula@uth.tmc.edu (V.K.C.); bela.patel@uth.tmc.edu (B.P.); bindu.h.akkanti@uth.tmc.edu (B.A.); 10 Division of Pulmonary, Critical Care, and Sleep Medicine, Department of Internal Medicine, McGovern Medical School at the University of Texas Health Science Center at Houston, 6431 Fannin St., MSB 1.150, Houston, TX 77030, USA

**Keywords:** cardiogenic shock, gastrointestinal bleeding, in-hospital mortality, ST-segment elevation myocardial infarction, National Inpatient Sample

## Abstract

**Background/Objectives**: Patients with ST-segment elevation myocardial infarction (STEMI) may develop gastrointestinal (GI) bleeding. We aimed to assess the impact of GI bleeding on in-hospital outcomes in patients with STEMI. **Methods**: This retrospective study was performed using the National Inpatient Sample from the United States of America from the years 2016–2021 to identify STEMI hospitalizations. Index admissions were stratified according to concomitant GI bleeding. The primary outcome was in-hospital mortality; secondary outcomes were inflation-adjusted total charges, total costs, length of stay ≥7 days, acute kidney injury, and cardiogenic shock. Multivariable analysis with a logistic regression model was used to identify associations with in-hospital mortality and several secondary outcomes. **Results**: Of 1,013,800 index admissions with STEMI, 22,260 (2.2%) had concomitant GI bleeding. Patients with GI bleeding were older and more frequently had comorbidities such as chronic heart failure, liver disease, and chronic kidney disease. In-hospital mortality was higher in patients with GI bleeding (27.8% versus 7.6%, *p* < 0.001). GI bleeding was associated with 2.30 times (2.11–2.51, *p* < 0.001), 3.78 times (3.50–4.07, *p* < 0.001), 2.99 times (2.76–3.24, *p* < 0.001), and 2.62 times (2.42–2.83, *p* < 0.001) increased odds of in-hospital mortality, length of stay ≥7 days, acute kidney injury, and cardiogenic shock, respectively. However, liver disease was most strongly positively associated with in-hospital mortality (odds ratio [OR]: 5.50 [5.21–5.80], *p* < 0.001), acute kidney injury (OR: 6.50 [6.18–6.84], *p* < 0.001), and cardiogenic shock (OR: 5.47 [5.22–5.74], *p* < 0.001). **Conclusions**: GI bleeding was associated with in-hospital mortality, adverse outcomes, and increased resource utilization in patients with STEMI, highlighting the need for risk stratification, implementation of preventative strategies, and timely treatment of GI bleeding.

## 1. Introduction

Acute coronary syndrome (ACS) encompasses a spectrum of clinical conditions characterized by certain patient signs and symptoms and potentially electrocardiogram and cardiac troponin changes [1]. ST-segment elevation myocardial infarction (STEMI), a subset of ACS, develops when an unstable atherosclerotic plaque ruptures or erodes, leading to complete occlusion of the coronary artery and transmural infarction [2]. Current guidelines in STEMI management recommend percutaneous coronary intervention (PCI) or thrombolysis as well as antiplatelet and anticoagulant agents [1,2]. Despite improvements in STEMI care—for example, with reperfusion times and overall defect-free care—over the past several years, risk-adjusted mortality has remained stable [3].

Major bleeding is a complication in patients with ACS or those undergoing PCI. Gastrointestinal (GI) bleeding is a common source of post-PCI bleeding [4]. It comprised 61.7% of post-discharge bleeding after PCI among those experiencing bleeding events during two-year follow-up [5]. While the incidence is overall low at approximately 0.9–1.1%, mortality increases significantly [6]. Furthermore, other concurrent or consequential cardiac conditions can increase the risk of bleeding; the highest risk of bleeding is associated with decompensated heart failure, cardiogenic shock, and mechanical circulatory support (MCS) rather than ACS and coronary revascularization [6].

Although studies have focused on GI bleeding, they included different patient populations, such as those undergoing PCI, those with an acute myocardial infarction (AMI), or those whose course was complicated by cardiogenic shock [7,8,9,10,11,12,13,14,15]. Fewer studies have targeted those with a STEMI [8]. We aimed to (1) compare the underlying characteristics of a cohort of patients with STEMI based on the presence of GI bleeding, (2) determine differences in resource utilization and complications between those with and without a GI bleed, and (3) identify associations of variables of interest, including GI bleeding, with in-hospital mortality, length of stay ≥7 days, acute kidney injury (AKI), and cardiogenic shock in all patients with STEMI. We used the National Inpatient Sample (NIS) from the United States of America (https://hcup-us.ahrq.gov/nisoverview.jsp [accessed 1 March 2026]) from 2016 to 2021, which encompasses over 1 million weighted STEMI hospitalizations.

## 2. Materials and Methods

### 2.1. Dataset and Patient Selection

The NIS is the largest, publicly available all-payer inpatient database under the Healthcare Cost and Utilization Project (HCUP), allowing for regional and national estimates to be obtained for resource utilization, quality, and outcomes. It includes information from 7 million and 35 million hospitalizations each year based on unweighted and weighted estimates, respectively. The NIS approximates a 20-percent stratified sample of all hospital discharges from community hospitals (non-federal, acute care hospitals). Further details regarding the NIS can be found elsewhere [16].

**Figure 1:**
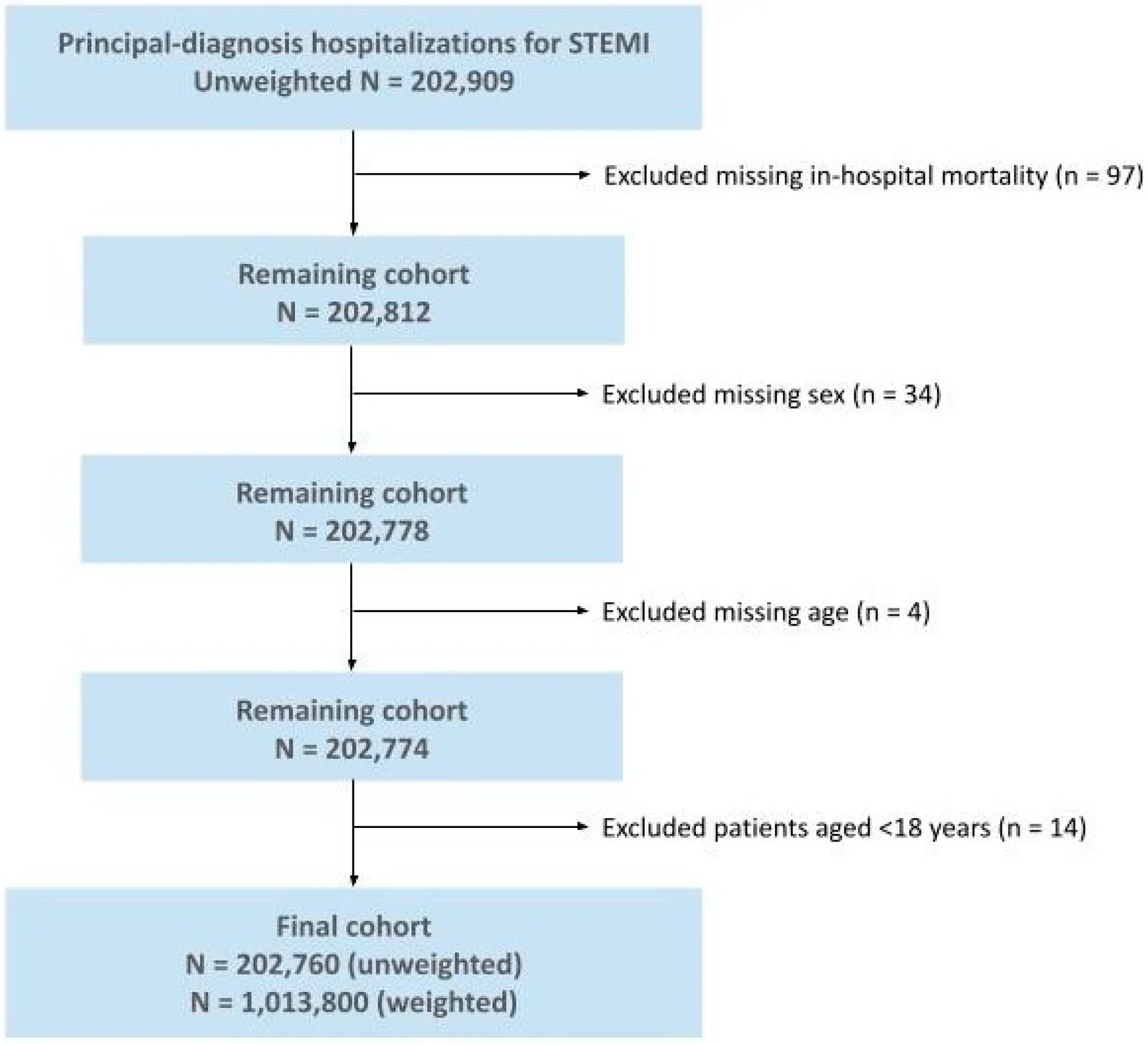
Cohort selection for the study. Hospitalizations with a principal diagnosis of an ST-segment elevation myocardial infarction (STEMI) were identified. Hospitalizations missing data on in-hospital mortality, age, and sex were excluded. Those that included patients less than 18 years of age were also excluded. The final sample size was 202,760 unweighted hospitalizations and 1,013,800 weighted hospitalizations.

For this retrospective study, we used the NIS from 2016–2021 to identify hospitalizations with a primary diagnosis of STEMI using International Classification of Diseases, Tenth Revision, Clinical Modification (ICD-10-CM) codes (see Table S1 and Figure 1). Hospitalizations that were missing data for in-hospital mortality (*n* = 97), sex (*n* = 34), and age (*n* = 4), and those including patients aged less than 18 years (*n* = 14) were excluded. Patients were further stratified according to the presence of GI bleeding, determined using ICD-10-CM codes in the secondary diagnosis position. Weighted estimates were obtained using the DISCWT variable provided by the HCUP, yielding 202,760 unweighted and 1,013,800 weighted hospitalizations in the final cohort. The number of hospitalizations across each calendar year can be found in Table S2. Institutional Review Board approval was not required given that the NIS is de-identified and publicly available [17].

### 2.2. Variables of Interest

Variables of interest included sex, age in years at admission, race/ethnicity, primary insurance (expected payer), region, location/teaching status, and bed size of the hospital, and the following comorbidities: hyperlipidemia, hypertension, chronic heart failure, prior myocardial infarction, prior PCI, prior coronary artery bypass grafting, obesity, smoker/tobacco user, chronic obstructive pulmonary disease, obstructive sleep apnea, prior stroke, liver disease (which encompassed conditions ranging from hepatitis from multiple etiologies to cirrhosis [see Table S1 for a full list of ICD-10-CM codes used]), chronic kidney disease/end-stage renal disease, diabetes mellitus, hypothyroidism, nutritional anemia, and GI bleeding.

### 2.3. Outcomes of Interest

The primary outcome was in-hospital mortality. Secondary outcomes were inflation-adjusted total charges (USD), total cost (USD), length of stay ≥7 days, AKI, and cardiogenic shock. AKI and cardiogenic shock were determined from ICD-10-CM codes as listed in Table S1. Although described as outcomes, AKI and cardiogenic shock were evaluated as coexisting diagnoses rather than causal outcomes or complications.

### 2.4. Statistical Analysis

Categorical variables are presented as frequency (percentage), and continuous variables are given as mean ± standard deviation. Baseline characteristics were compared using Pearson chi-square test and two-sample *t*-test for categorical and continuous variables, respectively. Similarly, primary and secondary outcomes were compared between the two groups using Pearson chi-square test for binary outcomes and two-sample *t*-test for continuous outcomes (i.e., inflation-adjusted total charges and total cost).

To identify associations of variables of interest with in-hospital mortality, multivariable analysis with a logistic regression model adjusted for demographics and covariates was performed. Multivariable analyses were performed using complete-case analysis, in which hospitalizations with missing values for any variable included in the respective model were excluded. Missing values were not imputed. To assess the robustness of our results regarding in-hospital mortality, we performed a sensitivity analysis running several logistic regression models: (1) excluding age group, (2) excluding sex, and (3) excluding race. Finally, we determined associations between variables of interest and length of stay ≥7 days, AKI, and cardiogenic shock with similar multivariable logistic regression analyses. Data regarding these associations are presented as adjusted odds ratios (95% confidence intervals) with the corresponding *p*-values.

All analyses incorporated the complex survey design of the NIS; discharge-level sampling weights were used to generate national estimates, while year-specific NIS strata and year-specific hospital identifiers were used to account for stratification and hospital-level clustering. A *p*-value <0.05 was considered statistically significant. Stata 18 (StataCorp, College Station, TX, USA) was used to perform all analyses.

## 3. Results

### 3.1. Baseline Characteristics

Of 1,013,800 index admissions for STEMI, 22,260 (2.2%) had concomitant GI bleeding (Table 1). The majority of patients were male, White, and had Medicare. Patients with GI bleeding were on average older (68.0 ± 12.7 years versus 63.5 ± 13.0 years, *p* < 0.001). The following conditions were more prevalent among those with STEMI and GI bleeding than those with STEMI but no GI bleeding: chronic heart failure (48.7% versus 25.5%), prior coronary artery bypass grafting (2.6% versus 2.0%), chronic obstructive pulmonary disease (18.4% versus 11.4%), prior stroke (8.1% versus 6.2%), liver disease (24.5% versus 5.0%), chronic kidney disease/end-stage renal disease (28.6% versus 13.6%), diabetes mellitus (35.9% versus 32.5%), and nutritional anemia (9.5% versus 2.0%). Baseline characteristics according to unweighted estimates are provided in Table S3.

### 3.2. Outcomes

Patients with STEMI and GI bleeding suffered more in-hospital mortality compared to those with STEMI only (27.8% versus 7.6%, *p* < 0.001) (Table 2). Among secondary outcomes, patients with STEMI and GI bleeding had higher adjusted total charges ($319,960.91 ± 497,572.85 versus $133,548.20 ± 158,507.56), higher total costs ($70,483.17 ± 100,385.55 versus $30,522.99 ± 31,264.34), and more frequent lengths of stay ≥7 days (49.8% versus 13.5%) (all *p* < 0.001). AKI (52.5% versus 16.8%, *p* < 0.001) and cardiogenic shock (42.9% versus 13.2%, *p* < 0.001) were both associated with GI bleeding.

### 3.3. Associations with In-Hospital Mortality

GI bleeding increased the odds of in-hospital mortality by 2.30 times (OR: 2.30 [2.11–2.51], *p* < 0.001) (Table 3). The strongest positive associations with in-hospital mortality were found with liver disease (OR: 5.50 [5.21–5.80], *p* < 0.001), followed by age ≥65 years compared to age 18 to less than 45 years (OR: 3.91 [3.51–4.36], *p* < 0.001) and GI bleeding. Sensitivity analyses yielded similar results (Table S4).

### 3.4. Associations with Secondary Outcomes

GI bleeding was associated with a length of stay ≥7 days (OR: 3.78 [3.50–4.07], *p* < 0.001), AKI (OR: 2.99 [2.76–3.24], *p* < 0.001), and cardiogenic shock (OR: 2.62 [2.42–2.83], *p* < 0.001) (Table 4). Chronic heart failure was most strongly associated with a length of stay ≥7 days (OR: 4.03 [3.91–4.16], *p* < 0.001). Liver disease was the variable of interest most strongly associated with AKI (OR: 6.50 [6.18–6.84], *p* < 0.001) and cardiogenic shock (OR: 5.47 [5.22–5.74], *p* < 0.001).

## 4. Discussion

GI bleeding can complicate STEMI hospitalizations. We found that among patients with STEMI, 2.2% had GI bleeding; these patients more frequently suffered from conditions such as chronic heart failure, chronic obstructive pulmonary disease, liver disease, and kidney disease. GI bleeding was associated with greater resource utilization and multiorgan involvement. The in-hospital mortality rate was higher among those with both GI bleeding and STEMI, with GI bleeding being independently associated with several adverse outcomes. However, liver disease had the strongest association with in-hospital death, AKI, and cardiogenic shock.

Similar to what has been previously described in the literature, we found characteristics that were associated with GI bleeding in the cohort. Using NIS data from 2003–2016, Albeiruti et al. found that those with STEMI and GI bleeding were older, female, and had a higher prevalence of pre-existing anemia, renal insufficiency, chronic lung disease, vascular disease, cirrhosis, atrial fibrillation/flutter, and cardiogenic shock [8]. In a smaller cohort of patients with STEMI, GI bleeding was associated with characteristics such as diabetes, history of bleeding, chronic kidney disease, peripheral vascular disease, recent surgery, previous coronary artery bypass grafting, cardiogenic shock, and intra-aortic balloon pump use [10]. Univariate [8] and multivariate [10] logistic regression models identified predictors of GI bleeding. Other studies explored associations with GI bleeding but with bleeding subtypes (e.g., upper GI bleeding) [13,15] or different patient populations (e.g., AMI or those undergoing PCI) [11,12,13]. Many of these conditions are associated with high bleeding risk [18].

While the proportion of GI bleeding was 2.2%, in-hospital mortality was 27.8% among those with GI bleeding and STEMI, and GI bleeding increased the odds of in-hospital mortality by 130%. We expand on existing evidence with an updated time period for capturing data, showing the continued burden of GI bleeding. The reported incidence of GI bleeding in STEMI was 2.2% from 2003–2016 via the NIS with a higher in-hospital mortality rate of 28.2% compared to our study [8]. The odds ratio for in-hospital mortality was lower at 1.91 ([1.85–1.97], *p* < 0.001) [8]. The incidence of GI bleeding in other studies ranged from 0.09% [11] to 22.9% [9] with variable incidences of in-hospital mortality [7,8,9,14]. Differences in the patient population studied and methods preclude direct comparisons in findings, but most studies have shown an association between GI bleeding and death across cohorts and follow-up periods [8,9,10,11,12,13,14,15,19]. The relationship between bleeding and mortality is likely multifactorial, including hemodynamic instability decreasing tissue perfusion, increased need for transfusions and the associated inflammation, interventions required to stabilize bleeding, and premature removal of antiplatelet agents, predisposing patients to recurrent ischemic events and thrombosis [4,20].

In this analysis, GI bleeding was associated with a higher prevalence of AKI and cardiogenic shock. The kidneys are sensitive to hemodynamic fluctuations. The reduction in arterial pressure with hemorrhagic shock results in decreased filtration pressure and perfusion, leading to tissue hypoxia; this is compounded by compensatory neurohormonal mechanisms that worsen hypoxia [21]. Although we did not assess cardiogenic shock, GI bleeding, and AKI together, cardiogenic shock is associated with a high prevalence of AKI, in part due to onset of cardiorenal syndrome, renal venous congestion and paradoxical effect of increased cardiac afterload for attempted renal protection [22]. Our study may have cases of mixed hemorrhagic and cardiogenic shock contributing to AKI. The association between cardiogenic shock and GI bleeding in patients with STEMI is also likely multifactorial, with mucosal compromise secondary to stress that is worsened by hemodynamic instability, reduced colonic perfusion leading to ischemic colitis, and concomitant use of antiplatelet and/or antithrombotic agents [6]. Additionally, MCS devices commonly used to treat cardiogenic shock may be at play [11,23,24]. Previous studies have identified a high likelihood of bleeding (up to 37%) for cardiogenic shock patients on MCS devices [25,26].

The complications arising from GI bleeding were reflected in increased resource utilization with higher inflation-adjusted total charges, total costs, and more frequent lengths of stay ≥7 days. GI bleeding was independently associated with a length of stay ≥7 days. Using data from the NIS between 2006–2012 to identify patients undergoing PCI, Patel et al. found that post-PCI GI bleeding was associated with an increase in length of stay by 4.23 days and cost of hospitalization by $9685 [12]. Similarly, Albeiruti et al. showed that GI bleeding in patients with STEMI was associated with increased non-home discharges, longer median lengths of stay, and higher costs in both unmatched and propensity score matched cohorts [8]. We only focused on the short-term costs of GI bleeding; resource utilization is likely to persist beyond the initial hospitalization [27]. Given these findings, early recognition, prevention, and management of GI bleeding are required to reduce healthcare burden and cost, such as with alternative dual antiplatelet therapy (DAPT) regimens such as shortening DAPT and DAPT de-escalation [1,2,4,28], proton pump inhibitor use [1,2], and use of endoscopy [6,8,29].

Although GI bleeding increased the odds of in-hospital mortality, liver disease was the most strongly associated with in-hospital mortality, AKI, and cardiogenic shock. Liver disease has been associated with worse outcomes in patients with cardiac conditions, including STEMI [30,31,32] and cardiogenic shock [33]. Among patients with nonalcoholic fatty liver disease, now known as metabolic dysfunction-associated steatotic liver disease, and STEMI, a higher grade or score of liver disease was associated with absent myocardial perfusion [30], increased major adverse cardiovascular events [30,31], and in-hospital mortality [30,31], and decreased long-term survival [31]. In an analysis of the Nationwide Readmissions Database, 4.4% of patients with STEMI who had cirrhosis or liver failure had increased odds of multiple complications, including in-hospital mortality, cardiogenic shock, major adverse cardiovascular events, post-procedural bleeding, need for transfusion, and increased resource utilization and all-cause readmission rates [32]. Among patients with cirrhosis and an AMI who received DAPT, cirrhosis was associated with higher 1-year mortality and increased GI bleeding, but lower recurrent AMI [34]. However, patients with decompensated cirrhosis who underwent left heart catheterization as part of liver transplant evaluation and were started on DAPT had no significant differences in GI bleeding, variceal hemorrhage, or 12-month mortality after GI bleeding compared to those without DAPT [35]. While seemingly conflicting, it does reflect a patient population that may not have ACS. Although our study and Kumar et al.’s did not assess the interaction between liver disease, GI bleeding, and in-hospital mortality in patients with STEMI, further studies should explore these relationships given the fragile, rebalanced hemostasis found in liver disease [36]. Prospective studies should examine antiplatelet and anticoagulation strategies in the high-risk subgroup of patients with cirrhosis with concurrent GI bleeding and STEMI, and further stratify them by the subtype of liver disease.

We showed that GI bleeding is associated with worse outcomes in patients with STEMI. However, there are limitations to this study. The NIS is an administrative database and is prone to selection, coding, and misclassification biases. Because the NIS focuses on discharge-level data, details on clinical factors such as days from STEMI to GI bleeding, antiplatelet and antithrombotic medications used, timing of AKI, and severity of cardiogenic shock could not be elucidated. The use of gastroprotective medications or specific antiplatelet/anticoagulation regimens could not be extracted. The degree of acute blood loss anemia could not be determined. The definition of liver disease was heterogeneous in our study. While liver disease was most strongly associated with in-hospital mortality, AKI, and cardiogenic shock among patients with STEMI, the degree of liver dysfunction was not explored. Further studies including these details will likely provide useful, nuanced insights. As with all observational studies, there were likely unmeasured confounders/residual confounding. Given the study design, causal relationships could not be established.

## 5. Conclusions

GI bleeding continues to be a serious, costly, and deadly complication in patients with STEMI. GI bleeding in STEMI highlights the dynamic interplay between multiple organ systems given associations with AKI and cardiogenic shock. Liver disease exemplifies the complexity in the management of patients with STEMI and identifies another potential high-risk group susceptible to adverse outcomes. Current evidence suggests adjusting the duration of antiplatelet therapy, using proton-pump inhibitors for prophylaxis, and thoughtfully using endoscopic evaluation as practical interventions that can reduce GI bleeding, improve patient outcomes, and alleviate the financial strain on the healthcare system. Ultimately, a proactive and personalized approach is necessary to optimize care in this high-risk population.

## Figures and Tables

**Table 1:** Descriptive statistics of patients with an ST-segment elevation myocardial infarction (STEMI), stratified according to the presence of gastrointestinal bleeding based on weighted estimates.

	STEMI with Gastrointestinal Bleeding	STEMI Without Gastrointestinal Bleeding	Total	*p*-Value
N	22,260 (2.2%)	991,540 (97.8%)	1,013,800 (100.0%)	
Sex				<0.001
Male	14,395 (64.7%)	691,155 (69.7%)	705,550 (69.6%)	
Female	7865 (35.3%)	300,385 (30.3%)	308,250 (30.4%)	
Age (years), mean ± standard deviation	68.0 ± 12.7	63.5 ± 13.0	63.6 ± 13.0	<0.001
Race/Ethnicity				<0.001
White	15,080 (71.0%)	715,935 (75.4%)	731,015 (75.3%)	
Black	2360 (11.1%)	83,755 (8.8%)	86,115 (8.9%)	
Hispanic	2115 (10.0%)	82,255 (8.7%)	84,370 (8.7%)	
Asian or Pacific Islander	820 (3.9%)	28,630 (3.0%)	29,450 (3.0%)	
Native American	115 (0.5%)	5195 (0.5%)	5310 (0.5%)	
Other	755 (3.6%)	34,180 (3.6%)	34,935 (3.6%)	
Primary insurance/expected payer				<0.001
Medicare	12,990 (58.4%)	440,750 (44.5%)	453,740 (44.8%)	
Medicaid	2465 (11.1%)	106,085 (10.7%)	108,550 (10.7%)	
Private insurance	4875 (21.9%)	336,320 (34.0%)	341,195 (33.7%)	
Self-pay	1175 (5.3%)	67,650 (6.8%)	68,825 (6.8%)	
No charge	115 (0.5%)	5565 (0.6%)	5680 (0.6%)	
Other	620 (2.8%)	33,310 (3.4%)	33,930 (3.4%)	
Region of hospital				0.071
Northeast	3920 (17.6%)	166,840 (16.8%)	170,760 (16.8%)	
Midwest	4940 (22.2%)	225,905 (22.8%)	230,845 (22.8%)	
South	8685 (39.0%)	401,045 (40.4%)	409,730 (40.4%)	
West	4715 (21.2%)	197,750 (19.9%)	202,465 (20.0%)	
Location/teaching status of hospital				<0.001
Rural	1100 (4.9%)	64,165 (6.5%)	65,265 (6.4%)	
Urban Non-Teaching	4175 (18.8%)	208,870 (21.1%)	213,045 (21.0%)	
Urban Teaching	16,985 (76.3%)	718,505 (72.5%)	735,490 (72.5%)	
Bed size of hospital				0.087
Small	3450 (15.5%)	163,415 (16.5%)	166,865 (16.5%)	
Medium	6470 (29.1%)	294,560 (29.7%)	301,030 (29.7%)	
Large	12,340 (55.4%)	533,565 (53.8%)	545,905 (53.8%)	
Comorbidities and conditions				
Hyperlipidemia	11,320 (50.9%)	648,160 (65.4%)	659,480 (65.1%)	<0.001
Hypertension	15,905 (71.5%)	728,370 (73.5%)	744,275 (73.4%)	0.003
Chronic heart failure	10,835 (48.7%)	252,455 (25.5%)	263,290 (26.0%)	<0.001
Prior myocardial infarction	2520 (11.3%)	120,400 (12.1%)	122,920 (12.1%)	0.098
Prior percutaneous coronary intervention	2355 (10.6%)	114,185 (11.5%)	116,540 (11.5%)	0.061
Prior coronary artery bypass grafting	575 (2.6%)	20,030 (2.0%)	20,605 (2.0%)	0.012
Obesity	3340 (15.0%)	183,385 (18.5%)	186,725 (18.4%)	<0.001
Smoker/tobacco user	9170 (41.2%)	514,780 (51.9%)	523,950 (51.7%)	<0.001
Chronic obstructive pulmonary disease	4085 (18.4%)	113,045 (11.4%)	117,130 (11.6%)	<0.001
Obstructive sleep apnea	1185 (5.3%)	59,760 (6.0%)	60,945 (6.0%)	0.059
Prior stroke	1800 (8.1%)	61,555 (6.2%)	63,355 (6.2%)	<0.001
Liver disease	5460 (24.5%)	49,095 (5.0%)	54,555 (5.4%)	<0.001
Chronic kidney disease/end-stage renal disease	6360 (28.6%)	134,830 (13.6%)	141,190 (13.9%)	<0.001
Diabetes mellitus	7995 (35.9%)	322,390 (32.5%)	330,385 (32.6%)	<0.001
Hypothyroidism	2085 (9.4%)	84,675 (8.5%)	86,760 (8.6%)	0.054
Nutritional anemia	2110 (9.5%)	20,265 (2.0%)	22,375 (2.2%)	<0.001

Descriptive statistics for patients with an ST-segment elevation myocardial infarction (STEMI), stratified by the presence of gastrointestinal bleeding based on weighted estimates. Categorical variables are given as frequencies (percentages) and continuous variables are given as mean ± standard deviation. Pearson chi-square test and two-sample *t*-tests were used to compare categorical and continuous variables, respectively.

**Table 2:** Comparison of outcomes in patients with an ST-segment elevation myocardial infarction (STEMI) according to the presence of gastrointestinal bleeding.

	STEMI with Gastrointestinal Bleeding	STEMI Without Gastrointestinal Bleeding	Total	*p*-Value
Primary outcome				
In-hospital mortality	6190 (27.8%)	75,075 (7.6%)	81,265 (8.0%)	<0.001
Secondary outcomes				
Inflation-adjusted total charges (USD), mean ± standard deviation	319,960.91± 497,572.85	133,548.20 ± 158,507.56	137,631.36 ± 175,329.77	<0.001
Total cost (USD), mean ± standard deviation	70,483.17 ± 100,385.55	30,522.99 ± 31,264.34	31,398.28 ± 34,798.58	<0.001
Length of stay ≥7 days	11,075 (49.8%)	133,955 (13.5%)	145,030 (14.3%)	<0.001
Acute kidney injury	11,695 (52.5%)	166,780 (16.8%)	178,475 (17.6%)	<0.001
Cardiogenic shock	9545 (42.9%)	131,175 (13.2%)	140,720 (13.9%)	<0.001

Primary and secondary outcomes in patients with an ST-segment elevation myocardial infarction (STEMI) with and without gastrointestinal bleeding. Pearson chi-square test was used to compare binary outcomes between both groups. Two-sample *t*-test was used to compare continuous outcomes (i.e., inflation-adjusted total charges and total cost). AKI and cardiogenic shock were considered as coexisting diagnoses rather than causal outcomes/complications.

**Table 3:** Associations of variables of interest with in-hospital mortality in all patients with an ST-segment elevation myocardial infarction.

Characteristic	Adjusted Odds Ratio (95% Confidence Interval)	*p*-Value
Sex		
Male	reference	—
Female	1.30 (1.25–1.35)	<0.001
Age group		
18 to less than 45 years	reference	—
45 to less than 65 years	1.69 (1.51–1.89)	<0.001
≥65	3.91 (3.51–4.36)	<0.001
Race/Ethnicity		
White	reference	—
Black	1.06 (1.00–1.14)	0.070
Hispanic	1.01 (0.95–1.08)	0.700
Asian or Pacific Islander	1.12 (1.01–1.23)	0.029
Native American	0.92 (0.72–1.17)	0.489
Other	1.22 (1.11–1.34)	<0.001
Gastrointestinal bleeding	2.30 (2.11–2.51)	<0.001
Comorbidities and conditions		
Hyperlipidemia	0.43 (0.41–0.44)	<0.001
Hypertension	0.75 (0.72–0.78)	<0.001
Chronic heart failure	1.51 (1.45–1.57)	<0.001
Prior myocardial infarction	0.91 (0.85–0.97)	0.002
Prior percutaneous coronary intervention	0.99 (0.93–1.06)	0.858
Prior coronary artery bypass grafting	1.21 (1.08–1.37)	0.001
Prior stroke	1.34 (1.26–1.44)	<0.001
Obesity	0.84 (0.79–0.88)	<0.001
Smoker/tobacco user	0.63 (0.61–0.66)	<0.001
Chronic obstructive pulmonary disease	1.40 (1.33–1.48)	<0.001
Obstructive sleep apnea	0.70 (0.64–0.77)	<0.001
Liver disease	5.50 (5.21–5.80)	<0.001
Chronic kidney disease/end-stage renal disease	1.60 (1.53–1.68)	<0.001
Diabetes mellitus	1.27 (1.22–1.33)	<0.001
Hypothyroidism	0.97 (0.91–1.03)	0.300
Nutritional anemia	0.70 (0.62–0.79)	<0.001

Associations between variables of interest and in-hospital mortality in all patients with an ST-segment elevation myocardial infarction were identified from multivariable logistic regression adjusted for demographics and covariates. Data are presented as adjusted odds ratios (95% confidence intervals) with the corresponding *p*-values.

**Table 4:** Associations of variables of interest with length of stay ≥7 days, acute kidney injury, or cardiogenic shock in all patients with an ST-segment elevation myocardial infarction.

	Length of Stay ≥7 Days		Acute Kidney Injury		Cardiogenic Shock	
Characteristic	Adjusted Odds Ratio (95% Confidence Interval)	*p*-Value	Adjusted Odds Ratio (95% Confidence Interval)	*p*-Value	Adjusted Odds Ratio (95% Confidence Interval)	*p*-Value
Sex						
Male	reference	—	reference	—	reference	—
Female	0.93 (0.90–0.96)	<0.001	0.73 (0.71–0.76)	<0.001	1.03 (0.99–1.06)	0.110
Age group						
18 to less than 45 years	reference	—	reference	—	reference	—
45 to less than 65 years	1.34 (1.25–1.43)	<0.001	1.17 (1.10–1.25)	<0.001	1.56 (1.46–1.68)	<0.001
≥65	1.64 (1.53–1.76)	<0.001	1.71 (1.60–1.82)	<0.001	2.06 (1.91–2.21)	<0.001
Race/Ethnicity						
White	reference	—	reference	—	reference	—
Black	1.13 (1.08–1.19)	<0.001	1.37 (1.30–1.44)	<0.001	0.85 (0.80–0.90)	<0.001
Hispanic	1.08 (1.03–1.14)	0.003	1.08 (1.03–1.14)	0.002	1.04 (0.99–1.09)	0.158
Asian or Pacific Islander	1.08 (1.00–1.18)	0.055	1.03 (0.96–1.12)	0.412	1.30 (1.21–1.41)	<0.001
Native American	1.08 (0.89–1.31)	0.455	0.87 (0.72–1.05)	0.147	1.21 (1.00–1.46)	0.054
Other	1.20 (1.11–1.30)	<0.001	1.15 (1.07–1.23)	<0.001	1.21 (1.12–1.30)	<0.001
Gastrointestinal bleeding	3.78 (3.50–4.07)	<0.001	2.99 (2.76–3.24)	<0.001	2.62 (2.42–2.83)	<0.001
Comorbidities and conditions						
Hyperlipidemia	0.80 (0.77–0.82)	<0.001	0.70 (0.68–0.73)	<0.001	0.65 (0.63–0.67)	<0.001
Hypertension	1.03 (0.99–1.06)	0.157	0.98 (0.94–1.01)	0.179	0.73 (0.71–0.76)	<0.001
Chronic heart failure	4.03 (3.91–4.16)	<0.001	2.52 (2.45–2.60)	<0.001	3.19 (3.10–3.29)	<0.001
Prior myocardial infarction	0.89 (0.85–0.93)	<0.001	0.89 (0.85–0.93)	<0.001	0.85 (0.81–0.90)	<0.001
Prior percutaneous coronary intervention	0.89 (0.84–0.93)	<0.001	0.91 (0.87–0.95)	<0.001	0.95 (0.91–1.00)	0.065
Prior coronary artery bypass grafting	0.81 (0.73–0.89)	<0.001	0.90 (0.82–0.99)	0.025	0.97 (0.88–1.07)	0.541
Prior stroke	1.13 (1.07–1.19)	<0.001	1.11 (1.05–1.17)	<0.001	1.00 (0.94–1.06)	0.990
Obesity	1.17 (1.13–1.22)	<0.001	1.16 (1.11–1.20)	<0.001	0.95 (0.92–0.99)	0.019
Smoker/tobacco user	0.77 (0.74–0.79)	<0.001	0.74 (0.72–0.77)	<0.001	0.82 (0.79–0.84)	<0.001
Chronic obstructive pulmonary disease	1.62 (1.55–1.69)	<0.001	1.16 (1.11–1.21)	<0.001	1.22 (1.17–1.28)	<0.001
Obstructive sleep apnea	0.99 (0.93–1.05)	0.729	0.91 (0.86–0.97)	0.003	0.80 (0.75–0.86)	<0.001
Liver disease	2.90 (2.76–3.05)	<0.001	6.50 (6.18–6.84)	<0.001	5.47 (5.22–5.74)	<0.001
Chronic kidney disease/end-stage renal disease	1.74 (1.67–1.80)	<0.001	5.70 (5.51–5.90)	<0.001	1.31 (1.26–1.37)	<0.001
Diabetes mellitus	1.25 (1.21–1.29)	<0.001	1.27 (1.23–1.31)	<0.001	1.16 (1.12–1.19)	<0.001
Hypothyroidism	0.95 (0.90–1.00)	0.042	1.02 (0.97–1.07)	0.405	0.93 (0.89–0.98)	0.007
Nutritional anemia	2.12 (1.96–2.29)	<0.001	1.48 (1.36–1.60)	<0.001	1.13 (1.04–1.24)	0.005

Associations between variables of interest and length of stay ≥7 days, acute kidney injury, or cardiogenic shock among all patients with an ST-segment elevation myocardial infarction. These associations were determined from multivariable logistic regression analysis adjusted for demographics and covariates. Data are presented as adjusted odds ratios (95% confidence intervals) with the corresponding *p*-values.

## Data Availability

The data supporting the conclusions of this paper are not available upon reasonable request due to mandates by the Healthcare Cost and Utilization Project.

## Supplementary Materials

The following supporting information can be downloaded at https://www.mdpi.com/article/10.3390/jcm15187140/s1, Table S1: International Classification of Diseases, Tenth Revision, Clinical Modification (ICD-10-CM) codes for selected characteristics used in the study; Table S2: Unweighted and weighted hospitalizations according to calendar year; Table S3: Descriptive statistics of patients with an ST-segment elevation myocardial infarction (STEMI), stratified according to the presence of gastrointestinal bleeding based on unweighted estimates; Table S4: Sensitivity analyses with exclusion of either age group, sex, or race from the model on associations with in-hospital mortality.

## Abbreviations

The following abbreviations are used in this manuscript:

ACS	Acute coronary syndrome
AKI	Acute kidney injury
AMI	Acute myocardial infarction
DAPT	Dual antiplatelet therapy
GI	Gastrointestinal
HCUP	Healthcare Cost and Utilization Project
ICD-10-CM	International Classification of Diseases, Tenth Revision, Clinical Modification
MCS	Mechanical circulatory support
NIS	National Inpatient Sample
PCI	Percutaneous coronary intervention
STEMI	ST-segment elevation myocardial infarction
