# Supplementary material for: Gastrointestinal Bleeding Is Associated with Worse In-Hospital Outcomes in Patients with ST-Segment Elevation Myocardial Infarction: An Analysis of the National Inpatient Sample
Source: J Clin Med. 2026 Sep 14;15(18):7140. doi: 10.3390/jcm15187140 (PMC13607591; doi:10.3390/jcm15187140)
Supplement: Supplementary file 1 [file jcm-15-07140-s001.zip › jcm-4433746-supplementary/Table S1.pdf]

Table S1: International Classification of Diseases, Tenth Revision, Clinical Modification (ICD-10-CM) codes for selected characteristics used in the study

Characteristic	ICD-10-CM codes
ST-segment elevation myocardial infarction	I2101, I2102, I2109, I2111, I2119, I2121, I2129, I213
Gastrointestinal bleeding	K2081, K2091, K2101, K2211, K250, K252, K254, K256, K260, K262, K264, K266, K270, K272, K274, K276, K280, K282, K284, K286, K2901, K2931, K2941, K2951, K2961, K2971, K2981, K2991, K31811, K3182, K50011, K50111, K50811, K50911, K51011, K51211, K51311, K51411, K51511, K51811, K51911, K5521, K5701, K5711, K5713, K5721, K5731, K5733, K5741, K5751, K5753, K5781, K5791, K5793, K625, K91841, K91871, K920, K921, K922, K9401, K9411, K9421, K9431
Acute kidney injury	N170, N171, N172, N178, N179, N19
Cardiogenic shock	R570
Liver disease	B180, B181, B182, B188, B189, K700, K7010, K7011, K702, K7030, K7031, K7040, K7041, K709, K710, K7110, K7111, K712, K713, K714, K7150, K7151, K716, K717, K718, K719, K7200, K7201, K7210, K7211, K7290, K7291, K730, K731, K732, K738, K739, K740, K7400, K7401, K7402, K741, K742, K743, K744, K745, K7460, K7469, K750, K751, K752, K753, K754, K7581, K7589, K759, K760, K761, K762, K763, K764, K765, K766, K767, K7681, K7689, K769, K77
