# Supplementary material for: Gastrointestinal Bleeding Is Associated with Worse In-Hospital Outcomes in Patients with ST-Segment Elevation Myocardial Infarction: An Analysis of the National Inpatient Sample
Source: J Clin Med. 2026 Sep 14;15(18):7140. doi: 10.3390/jcm15187140 (PMC13607591; doi:10.3390/jcm15187140)
Supplement: Supplementary file 1 [file jcm-15-07140-s001.zip › jcm-4433746-supplementary/Table S2.pdf]

Table S2: Unweighted and weighted hospitalizations according to calendar year

Year	Unweighted N	Weighted N
2016	35,223	176,115
2017	34,665	173,325
2018	33,699	168,495
2019	34,588	172,940
2020	31,970	159,850
2021	32,615	163,075
Total	202,760	1,013,800
