# Supplementary material for: Gastrointestinal Bleeding Is Associated with Worse In-Hospital Outcomes in Patients with ST-Segment Elevation Myocardial Infarction: An Analysis of the National Inpatient Sample
Source: J Clin Med. 2026 Sep 14;15(18):7140. doi: 10.3390/jcm15187140 (PMC13607591; doi:10.3390/jcm15187140)
Supplement: Supplementary file 1 [file jcm-15-07140-s001.zip › jcm-4433746-supplementary/Table S3.pdf]

Table S3: Descriptive statistics of patients with an ST-segment elevation myocardial infarction (STEMI), stratified according to the presence of gastrointestinal bleeding based on unweighted estimates

	STEMI with gastrointestinal bleeding	STEMI without gastrointestinal bleeding	Total	*p*-value
N	4,452 (2.2%)	198,308 (97.8%)	202,760 (100.0%)	
Sex				<0.001
Male	2,879 (64.7%)	138,231 (69.7%)	141,110 (69.6%)	
Female	1,573 (35.3%)	60,077 (30.3%)	61,650 (30.4%)	
Age (years), mean (standard deviation)	68.0±12.7	63.5±13.0	63.6±13.0	<0.001
Race/Ethnicity				<0.001
White	3,016 (71.0%)	143,187 (75.4%)	146,203 (75.3%)	
Black	472 (11.1%)	16,751 (8.8%)	17,223 (8.9%)	
Hispanic	423 (10.0%)	16,451 (8.7%)	16,874 (8.7%)	
Asian or Pacific Islander	164 (3.9%)	5,726 (3.0%)	5,890 (3.0%)	
Native American	23 (0.5%)	1,039 (0.5%)	1,062 (0.5%)	
Other	151 (3.6%)	6,836 (3.6%)	6,987 (3.6%)	
Primary insurance/expected payer				<0.001
Medicare	2,598 (58.4%)	88,150 (44.5%)	90,748 (44.8%)	
Medicaid	493 (11.1%)	21,217 (10.7%)	21,710 (10.7%)	
Private insurance	975 (21.9%)	67,264 (34.0%)	68,239 (33.7%)	
Self-pay	235 (5.3%)	13,530 (6.8%)	13,765 (6.8%)	
No charge	23 (0.5%)	1,113 (0.6%)	1,136 (0.6%)	
Other	124 (2.8%)	6,662 (3.4%)	6,786 (3.4%)	
Region of hospital				0.050
Northeast	784 (17.6%)	33,368 (16.8%)	34,152 (16.8%)	
Midwest	988 (22.2%)	45,181 (22.8%)	46,169 (22.8%)	
South	1,737 (39.0%)	80,209 (40.4%)	81,946 (40.4%)	
West	943 (21.2%)	39,550 (19.9%)	40,493 (20.0%)	
Location/teaching status of hospital				<0.001
Rural	220 (4.9%)	12,833 (6.5%)	13,053 (6.4%)	
Urban Non-Teaching	835 (18.8%)	41,774 (21.1%)	42,609 (21.0%)	
Urban Teaching	3,397 (76.3%)	143,701 (72.5%)	147,098 (72.5%)	
Bed size of hospital				0.071
Small	690 (15.5%)	32,683 (16.5%)	33,373 (16.5%)	
Medium	1,294 (29.1%)	58,912 (29.7%)	60,206 (29.7%)	
Large	2,468 (55.4%)	106,713 (53.8%)	109,181 (53.8%)	
Comorbidities and conditions				
Hyperlipidemia	2,264 (50.9%)	129,632 (65.4%)	131,896 (65.1%)	<0.001

Hypertension	3,181 (71.5%)	145,674 (73.5%)	148,855 (73.4%)	0.003
Chronic heart failure	2,167 (48.7%)	50,491 (25.5%)	52,658 (26.0%)	<0.001
Prior myocardial infarction	504 (11.3%)	24,080 (12.1%)	24,584 (12.1%)	0.097
Prior percutaneous coronary intervention	471 (10.6%)	22,837 (11.5%)	23,308 (11.5%)	0.053
Prior coronary artery bypass grafting	115 (2.6%)	4,006 (2.0%)	4,121 (2.0%)	0.008
Obesity	668 (15.0%)	36,677 (18.5%)	37,345 (18.4%)	<0.001
Smoker/Tobacco user	1,834 (41.2%)	102,956 (51.9%)	104,790 (51.7%)	<0.001
Chronic obstructive pulmonary disease	817 (18.4%)	22,609 (11.4%)	23,426 (11.6%)	<0.001
Obstructive sleep apnea	237 (5.3%)	11,952 (6.0%)	12,189 (6.0%)	0.051
Prior stroke	360 (8.1%)	12,311 (6.2%)	12,671 (6.2%)	<0.001
Liver disease	1,092 (24.5%)	9,819 (5.0%)	10,911 (5.4%)	<0.001
Chronic kidney disease/End-stage renal disease	1,272 (28.6%)	26,966 (13.6%)	28,238 (13.9%)	<0.001
Diabetes mellitus	1,599 (35.9%)	64,478 (32.5%)	66,077 (32.6%)	<0.001
Hypothyroidism	417 (9.4%)	16,935 (8.5%)	17,352 (8.6%)	0.051
Nutritional anemia	422 (9.5%)	4,053 (2.0%)	4,475 (2.2%)	<0.001

Descriptive statistics for patients with an ST-segment elevation myocardial infarction (STEMI), stratified by the presence of gastrointestinal bleeding based on unweighted estimates. Categorical variables are given as frequencies (percentages) and continuous variables are given as mean±standard deviation. Pearson chi-square test and two-sample t-tests were used to compare categorical and continuous variables, respectively.
