# Supplementary material for: Gastrointestinal Bleeding Is Associated with Worse In-Hospital Outcomes in Patients with ST-Segment Elevation Myocardial Infarction: An Analysis of the National Inpatient Sample
Source: J Clin Med. 2026 Sep 14;15(18):7140. doi: 10.3390/jcm15187140 (PMC13607591; doi:10.3390/jcm15187140)
Supplement: Supplementary file 1 [file jcm-15-07140-s001.zip › jcm-4433746-supplementary/Table S4.pdf]

Table S4: Sensitivity analyses with exclusion of either age group, sex, or race from the model on associations with in-hospital mortality

	Excluding age group		Excluding sex		Excluding race	
Characteristic	Adjusted odds ratio (95% confidence interval)	*p*-value	Adjusted odds ratio (95% confidence interval)	*p*-value	Adjusted odds ratio (95% confidence interval)	*p*-value
Sex						
Male	reference	—	—	—	reference	—
Female	1.45 (1.40-1.51)	<0.001	—	—	1.29 (1.24-1.34)	<0.001
Age group						
18 to less than 45 years	—	—	reference	—	reference	—
45 to less than 65 years	—	—	1.68 (1.51-1.88)	<0.001	1.67 (1.50-1.85)	<0.001
≥65	—	—	4.03 (3.61-4.49)	<0.001	3.85 (3.47-4.28)	<0.001
Race/Ethnicity						
White	reference	—	reference	—	—	—
Black	0.90 (0.84-0.96)	0.001	1.09 (1.02-1.16)	0.013	—	—
Hispanic	0.91 (0.86-0.97)	0.006	1.01 (0.94-1.07)	0.845	—	—
Asian or Pacific Islander	1.05 (0.95-1.16)	0.308	1.10 (1.00-1.22)	0.049	—	—
Native American	0.85 (0.66-1.08)	0.178	0.91 (0.71-1.17)	0.478	—	—
Other	1.11 (1.01-1.22)	0.029	1.21 (1.10-1.32)	<0.001	—	—
Gastrointestinal bleeding	2.40 (2.19-2.62)	<0.001	2.30 (2.10-2.51)	<0.001	2.32 (2.13-2.53)	<0.001
Comorbidities and conditions						
Hyperlipidemia	0.44 (0.43-0.46)	<0.001	0.42 (0.41-0.44)	<0.001	0.42 (0.41-0.44)	<0.001
Hypertension	0.83 (0.79-0.86)	<0.001	0.76 (0.73-0.79)	<0.001	0.74 (0.71-0.77)	<0.001
Chronic heart failure	1.58 (1.52-1.65)	<0.001	1.52 (1.46-1.58)	<0.001	1.51 (1.45-1.57)	<0.001
Prior myocardial infarction	0.91 (0.85-0.97)	0.002	0.90 (0.84-0.96)	0.001	0.90 (0.85-0.95)	0.001
Prior percutaneous coronary intervention	1.02 (0.96-1.09)	0.523	0.98 (0.92-1.04)	0.538	1.02 (0.96-1.09)	0.494
Prior coronary artery bypass grafting	1.35 (1.20-1.52)	<0.001	1.17 (1.04-1.32)	0.008	1.19 (1.06-1.34)	0.003

Prior stroke	1.47 (1.37-1.57)	<0.001	1.36 (1.28-1.46)	<0.001	1.33 (1.25-1.42)	<0.001
Obesity	0.72 (0.68-0.76)	<0.001	0.85 (0.81-0.90)	<0.001	0.84 (0.80-0.89)	<0.001
Smoker/tobacco user	0.55 (0.53-0.57)	<0.001	0.62 (0.60-0.65)	<0.001	0.63 (0.60-0.65)	<0.001
Chronic obstructive pulmonary disease	1.54 (1.46-1.63)	<0.001	1.43 (1.36-1.51)	<0.001	1.38 (1.31-1.45)	<0.001
Obstructive sleep apnea	0.69 (0.63-0.76)	<0.001	0.68 (0.62-0.74)	<0.001	0.69 (0.63-0.75)	<0.001
Liver disease	5.07 (4.81-5.34)	<0.001	5.43 (5.15-5.73)	<0.001	5.59 (5.30-5.89)	<0.001
Chronic kidney disease/End-stage renal disease	1.85 (1.76-1.94)	<0.001	1.58 (1.51-1.66)	<0.001	1.60 (1.52-1.67)	<0.001
Diabetes mellitus	1.25 (1.20-1.30)	<0.001	1.28 (1.23-1.33)	<0.001	1.28 (1.23-1.33)	<0.001
Hypothyroidism	1.07 (1.01-1.14)	0.034	1.04 (0.97-1.10)	0.266	0.95 (0.89-1.01)	0.081
Nutritional anemia	0.73 (0.65-0.82)	<0.001	0.72 (0.64-0.81)	<0.001	0.71 (0.63-0.80)	<0.001

A sensitivity analysis was performed excluding either age group, sex, or race from the model delineating associations between variables of interest with in-hospital mortality in all patients with an ST-segment elevation myocardial infarction. These were done using a multivariable logistic regression. Data are presented as adjusted odds ratio (95% confidence interval) with the associated *p*-values.
